# Cyclo­benzaprinium chloride

**DOI:** 10.1107/S1600536811024676

**Published:** 2011-06-30

**Authors:** M. S. Siddegowda, Jerry P. Jasinski, James A. Golen, H. S. Yathirajan, M. T. Swamy

**Affiliations:** a Department of Studies in Chemistry, University of Mysore, Manasagangotri, Mysore 570 006, India; b Department of Chemistry, Keene State College, 229 Main Street, Keene, NH 03435-2001, USA; c Department of Chemistry, Sambhram Institute of Technology, Bangalore 560 097, India

## Abstract

In the title mol­ecular salt [systematic name: 3-(5*H*-dibenzo[*a*,*d*]cyclo­hepten-5-yl­idene)-*N*,*N*-dimethyl­propan­aminium chloride], C_20_H_22_N^+^·Cl^−^, two cation–anion pairs make up the asymmetric unit. The dihedral angles between the mean planes of the two fused benzene rings of the cation are 49.5 (1) and 50.9 (1)°. The cystal packing is stabilized by N—H⋯Cl hydrogen bonds and weak C—H⋯Cl inter­actions.

## Related literature

For structurally related tricyclic anti­depressants, see: Cimolai (2009 ▶); Commissiong *et al.* (1981 ▶); Katz & Dube (1988 ▶). For related structures, see: Bindya *et al.* (2007 ▶); Fun *et al.* (2011 ▶); Portalone *et al.* (2007 ▶). For standard bond lengths, see Allen *et al.* (1987 ▶).
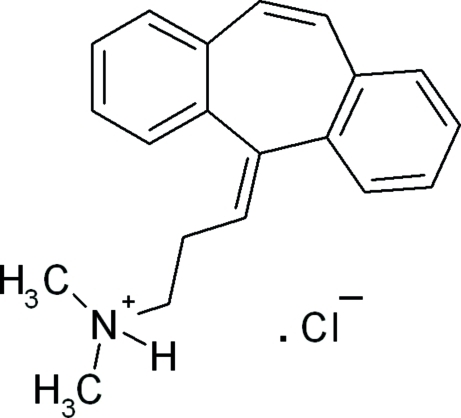


## Experimental

### 

#### Crystal data

C_20_H_22_N^+^·Cl^−^
*M* *_r_* = 311.84
Tetragonal, 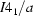

*a* = 32.0959 (7) Å
*c* = 13.7578 (5) Å
*V* = 14172.6 (7) Å^3^
*Z* = 32
Mo *K*α radiation
μ = 0.21 mm^−1^
*T* = 173 K
0.40 × 0.22 × 0.20 mm

#### Data collection

Oxford Diffraction Xcalibur Eos Gemini diffractometer
Absorption correction: multi-scan (*CrysAlis RED*; Oxford Diffraction, 2010 ▶) *T*_min_ = 0.920, *T*_max_ = 0.959
62421 measured reflections
8426 independent reflections
6210 reflections with *I* > 2σ(*I*)
*R*_int_ = 0.052

#### Refinement

*R*[*F* ^2^ > 2σ(*F* ^2^)] = 0.071
*wR*(*F* ^2^) = 0.198
*S* = 1.03
8426 reflections
407 parameters
2 restraints
H atoms treated by a mixture of independent and constrained refinement
Δρ_max_ = 1.00 e Å^−3^
Δρ_min_ = −0.38 e Å^−3^

### 

Data collection: *CrysAlis PRO* (Oxford Diffraction, 2010 ▶); cell refinement: *CrysAlis PRO*; data reduction: *CrysAlis RED* (Oxford Diffraction, 2010 ▶); program(s) used to solve structure: *SHELXS97* (Sheldrick, 2008 ▶); program(s) used to refine structure: *SHELXL97* (Sheldrick, 2008 ▶); molecular graphics: *SHELXTL* (Sheldrick, 2008 ▶); software used to prepare material for publication: *SHELXTL*.

## Supplementary Material

Crystal structure: contains datablock(s) global, I. DOI: 10.1107/S1600536811024676/jh2300sup1.cif

Structure factors: contains datablock(s) I. DOI: 10.1107/S1600536811024676/jh2300Isup2.hkl

Supplementary material file. DOI: 10.1107/S1600536811024676/jh2300Isup3.cml

Additional supplementary materials:  crystallographic information; 3D view; checkCIF report

## Figures and Tables

**Table 1:** Hydrogen-bond geometry (Å, °)

*D*—H⋯*A*	*D*—H	H⋯*A*	*D*⋯*A*	*D*—H⋯*A*
N1—H1*N*⋯Cl1	0.87 (2)	2.15 (2)	3.018 (2)	174 (2)
N2—H2*N*⋯Cl2	0.87 (2)	2.12 (2)	2.991 (2)	178 (3)
C19—H19*B*⋯Cl2^i^	0.98	2.83	3.702 (3)	149
C20—H20*B*⋯Cl2^i^	0.98	2.72	3.621 (3)	153
C38—H38*B*⋯Cl1	0.99	2.69	3.610 (3)	155

Symmetry code: (i) 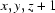.

**Fig. 1.**
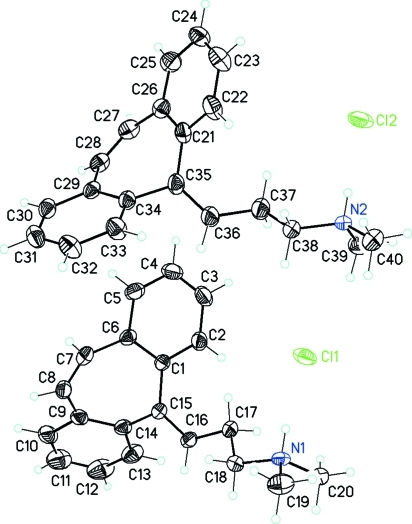
Molecular structure of the title compound showing two cation- anion pairs in the asymmetric unit, the atom labeling scheme and 50% probability displacement ellipsoids.

**Fig. 2.**
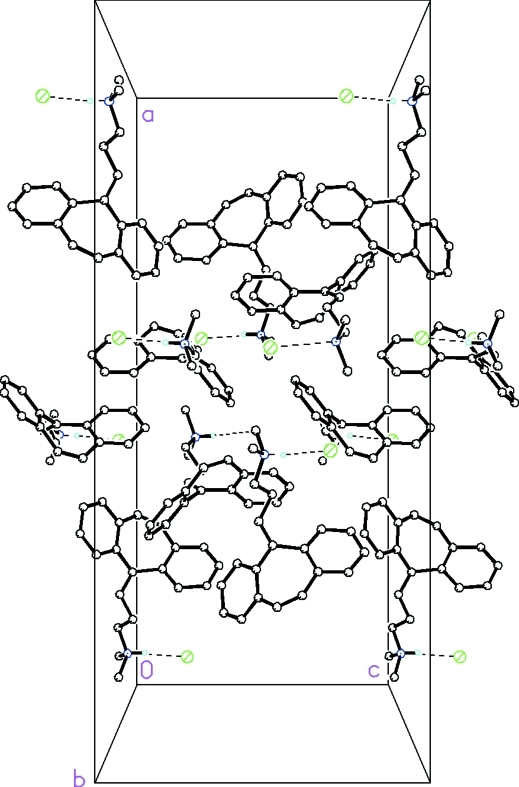
Packing diagram of the title compound viewed down the b axis. Dashed lines represent N—H···Cl hydrogen bonds and weak C—H···Cl intermolecular interactions.

## supplementary crystallographic information

### Comment

Cyclobenzaprine (Systematic iupac name: N,N-dimethyl-3-(dibenzo[a,d]cyclohepten-5-ylidene)propylamine) is a muscle relaxant medication used to relieve skeletal muscle spasms and associated pain in acute musculoskeletal conditions. It is the most well-studied drug for this application and it also has been used off-label for fibromyalgia treatment. Cyclobenzaprine has been considered structurally related to the first-generation tricyclic antidepressants (Commissiong *et al.*, 1981; Katz & Dube, 1988; Cimolai, 2009). The crystal structures of amitriptylinium picrate (Bindya *et al.*, 2007), 5-[3-(dimethylamino)propyl]-10,11-dihydro-5H-dibenz[a,d][7]annulen-5-ol (Portalone *et al.*, 2007) and cyclobenzaprinium salicylate (Fun *et al.*, 2011) have been reported. In view of the importance of yclobenzaprine, this paper reports the crystal structure of the title compound, C_20_H_22_N^+^. Cl^-^.

In the title molecular salt [Systematic name: 3-(5H-dibenzo[a,d]cyclohepten-5-ylidene)- N,N-dimethyl-1-propanamine hydrochloride], C_20_H_22_N^+^. Cl^-^, two cation-anion pairs crystallize in the asymmetric unit.  The dihedral angle between the mean planes of the two fused benzene rings of the cation are 49.5 (1)° and 50.9 (1)°, respectively.  Bond lengths are in normal positions (Allen *et al.*, 1987).  Cystal packing is stabilized by N—H···Cl hydrogen bonds and weak C—H···Cl intermolecular interactions (Fig. 2, Table 1).

### Experimental

The title compound was obtained as a gift sample from R. L. Fine Chem, Bangalore. X-ray quality crystals were obtained by slow evaporation of (1:1) methanol and dichloromethane  solution (m.p.: 458-460 K).

### Refinement

The N–H atoms were located by Fourier analysis and refined isotropically with DFIX = 0.86Å.  All of the remaining H atoms were placed in their calculated positions and then refined using the riding model with Atom–H lengths of 0.95Å (CH), 0.99Å (CH_2_) or 0.98Å (CH_3_).  Isotropic displacement parameters for these atoms were set to 1.19-1.21 (CH), 1.19-1.20 (CH_2_) or 1.49-1.51 (CH_3_) times *U*_eq_ of the parent atom.

### Crystal data

**Table:**

C_20_H_22_N^+^·Cl^−^	*D*_x_ = 1.169 Mg m^−^^3^
*M**_r_* = 311.84	Mo *K*α radiation, λ = 0.71073 Å
Tetragonal, *I*4_1_/*a*	Cell parameters from 11897 reflections
Hall symbol: -I 4ad	θ = 3.0–32.4°
*a* = 32.0959 (7) Å	µ = 0.21 mm^−^^1^
*c* = 13.7578 (5) Å	*T* = 173 K
*V* = 14172.6 (7) Å^3^	Block, colorless
*Z* = 32	0.40 × 0.22 × 0.20 mm
*F*(000) = 5312	

### Data collection

**Table:**

Oxford Diffraction Xcalibur Eos Gemini diffractometer	8426 independent reflections
Radiation source: Enhance (Mo) X-ray Source	6210 reflections with *I* > 2σ(*I*)
graphite	*R*_int_ = 0.052
Detector resolution: 16.1500 pixels mm^-1^	θ_max_ = 27.9°, θ_min_ = 3.0°
ω scans	*h* = −42→42
Absorption correction: multi-scan (*CrysAlis RED*; Oxford Diffraction, 2010)	*k* = −28→42
*T*_min_ = 0.920, *T*_max_ = 0.959	*l* = −18→18
62421 measured reflections	

### Refinement

**Table:**

Refinement on *F*^2^	Primary atom site location: structure-invariant direct methods
Least-squares matrix: full	Secondary atom site location: difference Fourier map
*R*[*F*^2^ > 2σ(*F*^2^)] = 0.071	Hydrogen site location: inferred from neighbouring sites
*wR*(*F*^2^) = 0.198	H atoms treated by a mixture of independent and constrained refinement
*S* = 1.03	*w* = 1/[σ^2^(*F*_o_^2^) + (0.0824*P*)^2^ + 20.447*P*] where *P* = (*F*_o_^2^ + 2*F*_c_^2^)/3
8426 reflections	(Δ/σ)_max_ = 0.004
407 parameters	Δρ_max_ = 1.00 e Å^−^^3^
2 restraints	Δρ_min_ = −0.38 e Å^−^^3^

### Special details

**Table:**

Geometry. All esds (except the esd in the dihedral angle between two l.s. planes) are estimated using the full covariance matrix. The cell esds are taken into account individually in the estimation of esds in distances, angles and torsion angles; correlations between esds in cell parameters are only used when they are defined by crystal symmetry. An approximate (isotropic) treatment of cell esds is used for estimating esds involving l.s. planes.
Refinement. Refinement of *F*^2^ against ALL reflections. The weighted *R*-factor *wR* and goodness of fit *S* are based on *F*^2^, conventional *R*-factors *R* are based on *F*, with *F* set to zero for negative *F*^2^. The threshold expression of *F*^2^ > σ(*F*^2^) is used only for calculating *R*-factors(gt) *etc*. and is not relevant to the choice of reflections for refinement. *R*-factors based on *F*^2^ are statistically about twice as large as those based on *F*, and *R*- factors based on ALL data will be even larger.

### Fractional atomic coordinates and isotropic or equivalent isotropic displacement parameters (Å^2^)

**Table:**

	*x*	*y*	*z*	*U*_iso_*/*U*_eq_	
Cl1	0.43755 (2)	0.83305 (3)	0.52604 (5)	0.0660 (2)	
Cl2	0.42392 (3)	0.82264 (3)	0.02610 (5)	0.0750 (3)	
N1	0.42875 (6)	0.83934 (6)	0.74395 (14)	0.0400 (4)	
H1N	0.4313 (8)	0.8397 (8)	0.6811 (13)	0.048*	
N2	0.43246 (7)	0.82088 (6)	0.24254 (15)	0.0457 (5)	
H2N	0.4305 (8)	0.8217 (9)	0.1797 (13)	0.055*	
C1	0.35291 (7)	0.97757 (6)	0.60833 (17)	0.0385 (5)	
C2	0.33019 (9)	0.95055 (8)	0.5497 (2)	0.0545 (7)	
H2A	0.3132	0.9298	0.5789	0.065*	
C3	0.33210 (12)	0.95355 (9)	0.4491 (2)	0.0730 (10)	
H3A	0.3158	0.9354	0.4100	0.088*	
C4	0.35725 (13)	0.98246 (10)	0.4061 (2)	0.0772 (10)	
H4A	0.3591	0.9839	0.3372	0.093*	
C5	0.37984 (10)	1.00939 (9)	0.4623 (2)	0.0614 (7)	
H5A	0.3977	1.0290	0.4317	0.074*	
C6	0.37719 (7)	1.00871 (7)	0.56414 (18)	0.0422 (5)	
C7	0.39916 (8)	1.04093 (7)	0.6178 (2)	0.0489 (6)	
H7A	0.4243	1.0506	0.5893	0.059*	
C8	0.38858 (9)	1.05853 (8)	0.7015 (2)	0.0523 (6)	
H8A	0.4063	1.0803	0.7236	0.063*	
C9	0.35311 (9)	1.04873 (8)	0.76403 (19)	0.0498 (6)	
C10	0.33717 (12)	1.08112 (10)	0.8215 (2)	0.0720 (9)	
H10A	0.3494	1.1080	0.8171	0.086*	
C11	0.30480 (15)	1.07497 (13)	0.8830 (3)	0.0934 (13)	
H11A	0.2942	1.0976	0.9202	0.112*	
C12	0.28720 (13)	1.03582 (14)	0.8918 (3)	0.0927 (13)	
H12A	0.2648	1.0315	0.9359	0.111*	
C13	0.30206 (10)	1.00289 (10)	0.8365 (2)	0.0681 (8)	
H13A	0.2897	0.9761	0.8427	0.082*	
C14	0.33494 (8)	1.00882 (7)	0.77176 (18)	0.0457 (5)	
C15	0.35139 (7)	0.97280 (7)	0.71591 (17)	0.0387 (5)	
C16	0.36159 (7)	0.93744 (7)	0.76229 (18)	0.0438 (5)	
H16A	0.3582	0.9375	0.8309	0.053*	
C17	0.37771 (8)	0.89783 (7)	0.71793 (18)	0.0445 (5)	
H17A	0.3867	0.9032	0.6502	0.053*	
H17B	0.3552	0.8767	0.7165	0.053*	
C18	0.41440 (7)	0.88136 (7)	0.77680 (17)	0.0427 (5)	
H18A	0.4379	0.9013	0.7716	0.051*	
H18B	0.4062	0.8797	0.8461	0.051*	
C19	0.47131 (8)	0.82985 (11)	0.7813 (2)	0.0649 (8)	
H19A	0.4805	0.8029	0.7559	0.097*	
H19B	0.4707	0.8288	0.8525	0.097*	
H19C	0.4907	0.8516	0.7601	0.097*	
C20	0.39931 (8)	0.80568 (8)	0.77229 (19)	0.0490 (6)	
H20A	0.3717	0.8115	0.7448	0.074*	
H20B	0.3973	0.8044	0.8433	0.074*	
H20C	0.4095	0.7789	0.7474	0.074*	
C21	0.44909 (8)	0.97348 (7)	0.1150 (2)	0.0497 (6)	
C22	0.47225 (10)	0.94587 (9)	0.0590 (2)	0.0668 (8)	
H22A	0.4893	0.9257	0.0903	0.080*	
C23	0.47100 (11)	0.94719 (11)	−0.0410 (3)	0.0756 (9)	
H23A	0.4879	0.9286	−0.0776	0.091*	
C24	0.44576 (11)	0.97486 (11)	−0.0883 (2)	0.0719 (9)	
H24A	0.4445	0.9751	−0.1573	0.086*	
C25	0.42190 (10)	1.00278 (10)	−0.0337 (2)	0.0628 (7)	
H25A	0.4038	1.0216	−0.0660	0.075*	
C26	0.42435 (7)	1.00330 (8)	0.0672 (2)	0.0492 (6)	
C27	0.40175 (8)	1.03607 (9)	0.1188 (2)	0.0549 (7)	
H27A	0.3764	1.0449	0.0897	0.066*	
C28	0.41211 (8)	1.05516 (8)	0.2012 (2)	0.0518 (6)	
H28A	0.3937	1.0765	0.2223	0.062*	
C29	0.44827 (7)	1.04757 (7)	0.26371 (18)	0.0428 (5)	
C30	0.46409 (8)	1.08065 (8)	0.3185 (2)	0.0525 (6)	
H30A	0.4511	1.1072	0.3141	0.063*	
C31	0.49824 (9)	1.07574 (9)	0.3793 (2)	0.0601 (7)	
H31A	0.5085	1.0988	0.4155	0.072*	
C32	0.51703 (10)	1.03762 (10)	0.3869 (2)	0.0656 (8)	
H32A	0.5406	1.0341	0.4277	0.079*	
C33	0.50154 (9)	1.00441 (9)	0.3350 (2)	0.0611 (7)	
H33A	0.5145	0.9780	0.3415	0.073*	
C34	0.46736 (8)	1.00845 (7)	0.27330 (19)	0.0457 (5)	
C35	0.45100 (8)	0.97120 (7)	0.2225 (2)	0.0509 (6)	
C36	0.43968 (11)	0.93803 (8)	0.2764 (2)	0.0648 (8)	
H36A	0.4439	0.9405	0.3445	0.078*	
C37	0.42116 (11)	0.89759 (8)	0.2417 (2)	0.0650 (8)	
H37A	0.3928	0.8941	0.2689	0.078*	
H37B	0.4190	0.8977	0.1700	0.078*	
C38	0.44836 (10)	0.86245 (8)	0.2740 (2)	0.0557 (7)	
H38A	0.4767	0.8666	0.2473	0.067*	
H38B	0.4505	0.8629	0.3458	0.067*	
C39	0.39106 (9)	0.80871 (10)	0.2797 (2)	0.0616 (7)	
H39A	0.3852	0.7798	0.2612	0.092*	
H39B	0.3698	0.8270	0.2517	0.092*	
H39C	0.3907	0.8112	0.3506	0.092*	
C40	0.46398 (9)	0.78921 (9)	0.2685 (2)	0.0611 (7)	
H40A	0.4552	0.7620	0.2437	0.092*	
H40B	0.4668	0.7878	0.3394	0.092*	
H40C	0.4908	0.7968	0.2397	0.092*	

### Atomic displacement parameters (Å^2^)

**Table:**

	*U*^11^	*U*^22^	*U*^33^	*U*^12^	*U*^13^	*U*^23^
Cl1	0.0768 (5)	0.0889 (5)	0.0323 (3)	−0.0323 (4)	0.0073 (3)	−0.0092 (3)
Cl2	0.1072 (6)	0.0865 (5)	0.0315 (3)	0.0333 (5)	−0.0039 (3)	−0.0032 (3)
N1	0.0440 (10)	0.0490 (11)	0.0271 (9)	0.0071 (8)	−0.0035 (8)	0.0039 (8)
N2	0.0686 (13)	0.0403 (10)	0.0282 (9)	−0.0061 (9)	0.0010 (9)	0.0022 (8)
C1	0.0407 (11)	0.0339 (10)	0.0408 (12)	0.0069 (8)	−0.0030 (9)	0.0031 (9)
C2	0.0655 (16)	0.0392 (12)	0.0590 (17)	0.0008 (11)	−0.0141 (13)	0.0002 (11)
C3	0.108 (3)	0.0513 (16)	0.0599 (19)	0.0113 (16)	−0.0318 (18)	−0.0139 (14)
C4	0.131 (3)	0.0618 (18)	0.0390 (15)	0.0235 (19)	−0.0056 (17)	−0.0026 (14)
C5	0.088 (2)	0.0496 (15)	0.0461 (15)	0.0133 (14)	0.0150 (14)	0.0085 (12)
C6	0.0472 (12)	0.0373 (11)	0.0423 (13)	0.0085 (9)	0.0037 (10)	0.0050 (9)
C7	0.0453 (13)	0.0427 (12)	0.0587 (16)	−0.0055 (10)	0.0012 (11)	0.0120 (11)
C8	0.0598 (15)	0.0400 (12)	0.0570 (16)	−0.0074 (11)	−0.0127 (12)	0.0039 (11)
C9	0.0662 (16)	0.0435 (13)	0.0398 (13)	0.0121 (11)	−0.0092 (11)	−0.0012 (10)
C10	0.114 (3)	0.0519 (16)	0.0507 (17)	0.0184 (16)	−0.0023 (18)	−0.0105 (13)
C11	0.136 (4)	0.080 (2)	0.065 (2)	0.045 (2)	0.020 (2)	−0.0065 (18)
C12	0.103 (3)	0.109 (3)	0.067 (2)	0.040 (2)	0.038 (2)	0.011 (2)
C13	0.0706 (19)	0.0690 (18)	0.0647 (19)	0.0136 (15)	0.0216 (16)	0.0120 (15)
C14	0.0522 (13)	0.0448 (12)	0.0401 (13)	0.0099 (10)	0.0016 (10)	0.0040 (10)
C15	0.0384 (11)	0.0362 (11)	0.0413 (12)	−0.0012 (8)	0.0017 (9)	0.0040 (9)
C16	0.0514 (13)	0.0386 (11)	0.0412 (13)	0.0004 (10)	0.0030 (10)	0.0060 (9)
C17	0.0537 (13)	0.0351 (11)	0.0448 (13)	0.0005 (10)	−0.0053 (11)	0.0037 (9)
C18	0.0488 (13)	0.0422 (12)	0.0370 (12)	−0.0015 (10)	−0.0033 (10)	−0.0009 (9)
C19	0.0447 (14)	0.089 (2)	0.0613 (18)	0.0136 (14)	−0.0100 (13)	0.0124 (16)
C20	0.0617 (15)	0.0421 (12)	0.0433 (14)	−0.0002 (11)	−0.0071 (11)	0.0013 (10)
C21	0.0515 (14)	0.0390 (12)	0.0587 (16)	−0.0020 (10)	−0.0109 (12)	−0.0065 (11)
C22	0.077 (2)	0.0534 (16)	0.071 (2)	0.0115 (14)	−0.0197 (16)	−0.0181 (14)
C23	0.080 (2)	0.076 (2)	0.070 (2)	0.0045 (17)	−0.0084 (17)	−0.0259 (17)
C24	0.091 (2)	0.077 (2)	0.0477 (17)	−0.0218 (18)	−0.0074 (16)	−0.0124 (15)
C25	0.0630 (17)	0.0684 (18)	0.0570 (17)	−0.0089 (14)	−0.0151 (14)	0.0049 (14)
C26	0.0426 (12)	0.0495 (13)	0.0554 (15)	−0.0066 (10)	−0.0074 (11)	0.0020 (11)
C27	0.0437 (13)	0.0607 (16)	0.0602 (17)	0.0093 (11)	−0.0023 (12)	0.0110 (13)
C28	0.0488 (14)	0.0474 (13)	0.0591 (17)	0.0120 (11)	0.0089 (12)	0.0089 (12)
C29	0.0429 (12)	0.0387 (11)	0.0468 (13)	0.0022 (9)	0.0110 (10)	0.0050 (10)
C30	0.0602 (15)	0.0387 (12)	0.0585 (16)	0.0013 (11)	0.0172 (13)	−0.0036 (11)
C31	0.0595 (16)	0.0569 (16)	0.0639 (18)	−0.0091 (13)	0.0107 (14)	−0.0197 (13)
C32	0.0589 (16)	0.0739 (19)	0.0639 (19)	0.0041 (14)	−0.0094 (14)	−0.0177 (15)
C33	0.0664 (17)	0.0521 (15)	0.0648 (18)	0.0157 (13)	−0.0142 (14)	−0.0078 (13)
C34	0.0504 (13)	0.0377 (11)	0.0490 (14)	0.0020 (10)	−0.0021 (11)	0.0003 (10)
C35	0.0593 (15)	0.0346 (11)	0.0588 (16)	0.0054 (10)	−0.0136 (12)	−0.0018 (11)
C36	0.095 (2)	0.0423 (14)	0.0573 (17)	0.0042 (14)	−0.0209 (16)	0.0002 (12)
C37	0.083 (2)	0.0470 (15)	0.0652 (19)	−0.0013 (14)	−0.0148 (16)	0.0026 (13)
C38	0.0817 (19)	0.0412 (13)	0.0441 (14)	−0.0082 (12)	−0.0023 (13)	0.0028 (11)
C39	0.0633 (17)	0.0662 (17)	0.0553 (17)	−0.0059 (13)	0.0060 (13)	0.0006 (14)
C40	0.0643 (17)	0.0545 (15)	0.0643 (19)	0.0005 (13)	0.0015 (14)	0.0099 (13)

### Geometric parameters (Å, °)

**Table:**

N1—C20	1.487 (3)	C19—H19C	0.9800
N1—C19	1.491 (3)	C20—H20A	0.9800
N1—C18	1.495 (3)	C20—H20B	0.9800
N1—H1N	0.869 (17)	C20—H20C	0.9800
N2—C39	1.476 (4)	C21—C22	1.390 (4)
N2—C40	1.478 (4)	C21—C26	1.407 (4)
N2—C38	1.493 (3)	C21—C35	1.483 (4)
N2—H2N	0.867 (17)	C22—C23	1.377 (5)
C1—C2	1.391 (3)	C22—H22A	0.9500
C1—C6	1.406 (3)	C23—C24	1.367 (5)
C1—C15	1.489 (3)	C23—H23A	0.9500
C2—C3	1.389 (4)	C24—C25	1.397 (5)
C2—H2A	0.9500	C24—H24A	0.9500
C3—C4	1.365 (5)	C25—C26	1.391 (4)
C3—H3A	0.9500	C25—H25A	0.9500
C4—C5	1.368 (5)	C26—C27	1.461 (4)
C4—H4A	0.9500	C27—C28	1.331 (4)
C5—C6	1.404 (4)	C27—H27A	0.9500
C5—H5A	0.9500	C28—C29	1.465 (4)
C6—C7	1.453 (4)	C28—H28A	0.9500
C7—C8	1.327 (4)	C29—C30	1.398 (4)
C7—H7A	0.9500	C29—C34	1.403 (3)
C8—C9	1.461 (4)	C30—C31	1.387 (4)
C8—H8A	0.9500	C30—H30A	0.9500
C9—C10	1.403 (4)	C31—C32	1.368 (4)
C9—C14	1.411 (4)	C31—H31A	0.9500
C10—C11	1.354 (5)	C32—C33	1.375 (4)
C10—H10A	0.9500	C32—H32A	0.9500
C11—C12	1.383 (6)	C33—C34	1.394 (4)
C11—H11A	0.9500	C33—H33A	0.9500
C12—C13	1.387 (5)	C34—C35	1.481 (3)
C12—H12A	0.9500	C35—C36	1.347 (4)
C13—C14	1.394 (4)	C36—C37	1.505 (4)
C13—H13A	0.9500	C36—H36A	0.9500
C14—C15	1.485 (3)	C37—C38	1.494 (4)
C15—C16	1.343 (3)	C37—H37A	0.9900
C16—C17	1.502 (3)	C37—H37B	0.9900
C16—H16A	0.9500	C38—H38A	0.9900
C17—C18	1.524 (3)	C38—H38B	0.9900
C17—H17A	0.9900	C39—H39A	0.9800
C17—H17B	0.9900	C39—H39B	0.9800
C18—H18A	0.9900	C39—H39C	0.9800
C18—H18B	0.9900	C40—H40A	0.9800
C19—H19A	0.9800	C40—H40B	0.9800
C19—H19B	0.9800	C40—H40C	0.9800
			
C20—N1—C19	110.1 (2)	N1—C20—H20A	109.5
C20—N1—C18	112.34 (19)	N1—C20—H20B	109.5
C19—N1—C18	111.3 (2)	H20A—C20—H20B	109.5
C20—N1—H1N	109.2 (17)	N1—C20—H20C	109.5
C19—N1—H1N	105.1 (17)	H20A—C20—H20C	109.5
C18—N1—H1N	108.6 (17)	H20B—C20—H20C	109.5
C39—N2—C40	110.5 (2)	C22—C21—C26	118.5 (3)
C39—N2—C38	116.3 (2)	C22—C21—C35	120.0 (2)
C40—N2—C38	108.1 (2)	C26—C21—C35	121.6 (2)
C39—N2—H2N	106.7 (19)	C23—C22—C21	121.3 (3)
C40—N2—H2N	108.2 (19)	C23—C22—H22A	119.4
C38—N2—H2N	106.6 (19)	C21—C22—H22A	119.4
C2—C1—C6	118.9 (2)	C24—C23—C22	120.8 (3)
C2—C1—C15	119.7 (2)	C24—C23—H23A	119.6
C6—C1—C15	121.4 (2)	C22—C23—H23A	119.6
C3—C2—C1	120.7 (3)	C23—C24—C25	119.1 (3)
C3—C2—H2A	119.6	C23—C24—H24A	120.5
C1—C2—H2A	119.6	C25—C24—H24A	120.5
C4—C3—C2	120.4 (3)	C26—C25—C24	120.9 (3)
C4—C3—H3A	119.8	C26—C25—H25A	119.6
C2—C3—H3A	119.8	C24—C25—H25A	119.6
C5—C4—C3	119.8 (3)	C25—C26—C21	119.4 (3)
C5—C4—H4A	120.1	C25—C26—C27	117.8 (3)
C3—C4—H4A	120.1	C21—C26—C27	122.9 (3)
C4—C5—C6	121.5 (3)	C28—C27—C26	128.4 (2)
C4—C5—H5A	119.2	C28—C27—H27A	115.8
C6—C5—H5A	119.2	C26—C27—H27A	115.8
C5—C6—C1	118.4 (2)	C27—C28—C29	128.4 (2)
C5—C6—C7	117.8 (2)	C27—C28—H28A	115.8
C1—C6—C7	123.7 (2)	C29—C28—H28A	115.8
C8—C7—C6	128.3 (2)	C30—C29—C34	118.1 (2)
C8—C7—H7A	115.9	C30—C29—C28	118.6 (2)
C6—C7—H7A	115.9	C34—C29—C28	123.4 (2)
C7—C8—C9	128.2 (2)	C31—C30—C29	121.7 (2)
C7—C8—H8A	115.9	C31—C30—H30A	119.1
C9—C8—H8A	115.9	C29—C30—H30A	119.1
C10—C9—C14	118.7 (3)	C32—C31—C30	119.7 (3)
C10—C9—C8	117.2 (3)	C32—C31—H31A	120.1
C14—C9—C8	124.2 (2)	C30—C31—H31A	120.1
C11—C10—C9	121.6 (3)	C31—C32—C33	119.6 (3)
C11—C10—H10A	119.2	C31—C32—H32A	120.2
C9—C10—H10A	119.2	C33—C32—H32A	120.2
C10—C11—C12	120.0 (3)	C32—C33—C34	121.9 (3)
C10—C11—H11A	120.0	C32—C33—H33A	119.0
C12—C11—H11A	120.0	C34—C33—H33A	119.0
C11—C12—C13	120.3 (3)	C33—C34—C29	119.0 (2)
C11—C12—H12A	119.9	C33—C34—C35	119.4 (2)
C13—C12—H12A	119.9	C29—C34—C35	121.5 (2)
C12—C13—C14	120.5 (3)	C36—C35—C34	118.3 (3)
C12—C13—H13A	119.8	C36—C35—C21	125.2 (2)
C14—C13—H13A	119.8	C34—C35—C21	116.5 (2)
C13—C14—C9	119.0 (2)	C35—C36—C37	127.9 (3)
C13—C14—C15	119.6 (2)	C35—C36—H36A	116.1
C9—C14—C15	121.4 (2)	C37—C36—H36A	116.1
C16—C15—C14	119.9 (2)	C38—C37—C36	109.0 (3)
C16—C15—C1	123.5 (2)	C38—C37—H37A	109.9
C14—C15—C1	116.48 (19)	C36—C37—H37A	109.9
C15—C16—C17	127.3 (2)	C38—C37—H37B	109.9
C15—C16—H16A	116.3	C36—C37—H37B	109.9
C17—C16—H16A	116.3	H37A—C37—H37B	108.3
C16—C17—C18	110.1 (2)	N2—C38—C37	112.9 (2)
C16—C17—H17A	109.6	N2—C38—H38A	109.0
C18—C17—H17A	109.6	C37—C38—H38A	109.0
C16—C17—H17B	109.6	N2—C38—H38B	109.0
C18—C17—H17B	109.6	C37—C38—H38B	109.0
H17A—C17—H17B	108.2	H38A—C38—H38B	107.8
N1—C18—C17	112.98 (19)	N2—C39—H39A	109.5
N1—C18—H18A	109.0	N2—C39—H39B	109.5
C17—C18—H18A	109.0	H39A—C39—H39B	109.5
N1—C18—H18B	109.0	N2—C39—H39C	109.5
C17—C18—H18B	109.0	H39A—C39—H39C	109.5
H18A—C18—H18B	107.8	H39B—C39—H39C	109.5
N1—C19—H19A	109.5	N2—C40—H40A	109.5
N1—C19—H19B	109.5	N2—C40—H40B	109.5
H19A—C19—H19B	109.5	H40A—C40—H40B	109.5
N1—C19—H19C	109.5	N2—C40—H40C	109.5
H19A—C19—H19C	109.5	H40A—C40—H40C	109.5
H19B—C19—H19C	109.5	H40B—C40—H40C	109.5
			
C6—C1—C2—C3	1.5 (4)	C26—C21—C22—C23	−0.5 (4)
C15—C1—C2—C3	−178.0 (2)	C35—C21—C22—C23	179.8 (3)
C1—C2—C3—C4	1.7 (5)	C21—C22—C23—C24	−2.2 (5)
C2—C3—C4—C5	−1.8 (5)	C22—C23—C24—C25	1.7 (5)
C3—C4—C5—C6	−1.4 (5)	C23—C24—C25—C26	1.5 (5)
C4—C5—C6—C1	4.5 (4)	C24—C25—C26—C21	−4.2 (4)
C4—C5—C6—C7	−174.3 (3)	C24—C25—C26—C27	173.9 (3)
C2—C1—C6—C5	−4.5 (3)	C22—C21—C26—C25	3.7 (4)
C15—C1—C6—C5	175.0 (2)	C35—C21—C26—C25	−176.7 (2)
C2—C1—C6—C7	174.3 (2)	C22—C21—C26—C27	−174.4 (3)
C15—C1—C6—C7	−6.3 (3)	C35—C21—C26—C27	5.3 (4)
C5—C6—C7—C8	147.6 (3)	C25—C26—C27—C28	−146.9 (3)
C1—C6—C7—C8	−31.2 (4)	C21—C26—C27—C28	31.2 (4)
C6—C7—C8—C9	3.3 (5)	C26—C27—C28—C29	−2.0 (5)
C7—C8—C9—C10	−151.7 (3)	C27—C28—C29—C30	151.2 (3)
C7—C8—C9—C14	30.0 (4)	C27—C28—C29—C34	−30.4 (4)
C14—C9—C10—C11	−0.5 (5)	C34—C29—C30—C31	1.5 (4)
C8—C9—C10—C11	−178.9 (3)	C28—C29—C30—C31	180.0 (2)
C9—C10—C11—C12	1.3 (6)	C29—C30—C31—C32	−0.4 (4)
C10—C11—C12—C13	−1.2 (7)	C30—C31—C32—C33	−0.8 (5)
C11—C12—C13—C14	0.2 (6)	C31—C32—C33—C34	0.9 (5)
C12—C13—C14—C9	0.5 (5)	C32—C33—C34—C29	0.1 (5)
C12—C13—C14—C15	177.8 (3)	C32—C33—C34—C35	−177.5 (3)
C10—C9—C14—C13	−0.4 (4)	C30—C29—C34—C33	−1.3 (4)
C8—C9—C14—C13	177.8 (3)	C28—C29—C34—C33	−179.7 (3)
C10—C9—C14—C15	−177.6 (2)	C30—C29—C34—C35	176.2 (2)
C8—C9—C14—C15	0.7 (4)	C28—C29—C34—C35	−2.2 (4)
C13—C14—C15—C16	−50.4 (4)	C33—C34—C35—C36	55.9 (4)
C9—C14—C15—C16	126.7 (3)	C29—C34—C35—C36	−121.6 (3)
C13—C14—C15—C1	125.7 (3)	C33—C34—C35—C21	−123.1 (3)
C9—C14—C15—C1	−57.2 (3)	C29—C34—C35—C21	59.3 (3)
C2—C1—C15—C16	56.8 (3)	C22—C21—C35—C36	−61.0 (4)
C6—C1—C15—C16	−122.7 (3)	C26—C21—C35—C36	119.3 (3)
C2—C1—C15—C14	−119.1 (2)	C22—C21—C35—C34	118.0 (3)
C6—C1—C15—C14	61.4 (3)	C26—C21—C35—C34	−61.7 (3)
C14—C15—C16—C17	178.6 (2)	C34—C35—C36—C37	178.0 (3)
C1—C15—C16—C17	2.9 (4)	C21—C35—C36—C37	−3.0 (5)
C15—C16—C17—C18	134.8 (3)	C35—C36—C37—C38	122.6 (3)
C20—N1—C18—C17	−73.1 (2)	C39—N2—C38—C37	−61.3 (3)
C19—N1—C18—C17	163.0 (2)	C40—N2—C38—C37	173.7 (3)
C16—C17—C18—N1	173.04 (19)	C36—C37—C38—N2	−179.8 (3)

### Hydrogen-bond geometry (Å, °)

**Table:**

*D*—H···*A*	*D*—H	H···*A*	*D*···*A*	*D*—H···*A*
N1—H1N···Cl1	0.87 (2)	2.15 (2)	3.018 (2)	174 (2)
N2—H2N···Cl2	0.87 (2)	2.12 (2)	2.991 (2)	178 (3)
C19—H19B···Cl2^i^	0.98	2.83	3.702 (3)	149
C20—H20B···Cl2^i^	0.98	2.72	3.621 (3)	153
C38—H38B···Cl1	0.99	2.69	3.610 (3)	155

Symmetry codes: (i) *x*, *y*, *z*+1.
